# Photobiomodulation enhances facial nerve regeneration via activation of PI3K/Akt signaling pathway–mediated antioxidant response

**DOI:** 10.1007/s10103-021-03344-8

**Published:** 2021-07-24

**Authors:** Bohan Li, Xiao Wang

**Affiliations:** Department of Stomatology, Peking University Third Hospital, No. 49 North Garden Road, Haidian District, Beijing, 100191 China

**Keywords:** Photobiomodulation, Facial nerve regeneration, PI3K/Akt signaling pathway, Antioxidant response, Schwann cells

## Abstract

Facial nerve dysfunction is a common clinical condition that leads to disfigurement and emotional distress in the affected individuals. This study aimed to evaluate whether photobiomodulation can enhance regeneration of crushed facial nerves and attempt to investigate the possible underlying mechanism of neuroprotective function and therapeutic target. Various parameters of photobiomodulation were assigned to the facial nerves and Schwann cells (SCs) separately during crushed injury in rats. Axonal regeneration, functional outcomes, and SC apoptosis, proliferation, and underlying mechanisms of action were evaluated by morphological, histopathological, and functional assessments, flow cytometry, western blotting, real-time PCR, and IncuCyte. The results showed that photobiomodulation improved axonal regeneration and functional recovery, and also promoted proliferation, and inhibited apoptosis of SCs, both of these were considered as the most effective parameters in 250mW group. In addition, the neuroprotective effects of photobiomodulation (500mW) were likely associated with oxidative stress–induced SC apoptosis via activation of the PI3K/Akt signaling pathway. Our results revealed that photobiomodulation significantly promoted axonal regeneration, functional recovery, and regeneration of the facial nucleus, and its mechanism was related to the up-regulation of the PI3K/Akt signaling pathway. These findings provide clear experimental evidence of photobiomodulation as an alternative therapeutic strategy for peripheral nerve damage.

## Background

Facial nerve injury combined with the loss of facial expression is a health problem that can significantly deteriorate quality of life (Yasui et al. 2016). Such a condition can seriously hinder the verbal communication conveyed by facial expressions that are considered essential to social relationships. For example, a spontaneous and dynamic smile has a great impact on social interactions (Seo et al. 2018; Lee et al. 2017; Li et al. 2012).

Schwann cells (SCs) have an essential role in axon remyelination. SCs form myelin sheath and maintain its integrity in the peripheral nervous system (PNS) (Shibeeb et al. 2014). SCs can be immediately activated and dedifferentiated during PNS injury to repair the cells, which subsequently stimulates the severed axons to promote axon regeneration (Hou et al. 2018; Wang et al. 2018; Mao et al. 2018; Quintes and Brinkmann 2017).

Numerous in vivo and in vitro studies have shown that biostimulation (i.e., laser treatment and electrical stimulation) promotes adhesion, growth, proliferation, and differentiation of various cells, including neural stem cells, SCs, embryonic stem cells, pluripotent stem cells, and mesenchymal stem cells (Shibeeb et al. 2014; Pouriran et al. 2016; Rimington et al. 2018; Cakir et al. 2016). Previous studies have also revealed that SCs continuously promote the increase of neurite growth under photobiomodulation with a variety of parameters, which in turn could modulate the mobility and differentiation of neural cells. In addition, functional, histopathological, morphological, and electrophysiological assessment of photobiomodulation proved its beneficial effects on the regeneration of peripheral nerves following an injury (Yazdani et al. 2012).

According to the previous literature, the phosphatidylinositol-3 kinase/protein kinase B (PI3K/Akt) signaling pathway has an important role in reducing the survival, proliferation, axon growth, and myelin sheath of SCs (Huang et al. 2017; Dong et al. 2019). Huang et al. (2017) reported that PI3K/Akt signaling pathway can accelerate and provide resistance to oxidative stress in neurons. Our data revealed that photobiomodulation has a neuroprotective effect in improving angiogenesis and providing resistance to apoptosis. The purpose of this study was to investigate whether photobiomodulation has a neuroprotective role in facilitating axonal regeneration and functional recovery, and to furthermore explore potentially related molecular mechanisms.

## Materials and methods

### Animals and crush injury

Eight-week-old male Sprague–Dawley (SD) rats weighing 200–250 g were purchased from an animal supplier (KeYu Co., China) (SCXK (Jing) 2016–0002). The animal permission number was XYXK (Jing) 2015–0007. The experiments were performed after adaptation in the animal facility for 1 week.

SD rats were maintained in rooms at constant temperature (22 °C) and humidity (50%). The protocol for conducting animal experiments was approved by the Committee for Animal Experimentation of the 309 Hospital of PLA, China (CNU IACUC-H-2013–16). Rats were anesthetized via intraperitoneal injection of Zolletil® (a combination of tiletamine and zolazepam in 1:1 ratio, Virbac, Carros, France) and xylazine hydrochloride. A post-auricular incision was made on the left side of each rat. The main trunk of the facial nerve was exposed, passed through the stylomastoid foramen, and fixed before branching of the main trunk. The entire hemifacial movements were identified by electrical stimulation of the main trunk. The 5 mm length nerve was crushed with hemostatic forceps for 30 s from its origin to cause a facial nerve crush defect.

### Laser treatment protocol

In this study, we used a diode laser (Daheng New Epoch Technology, China). The protocol was applied transcutaneously to three points along the facial nerve on the skin surface at a wavelength of 980 nm. Laser therapy was applied from the day after surgery till the postoperative period three times per week for 5 consecutive weeks.

**Fig. 1:**
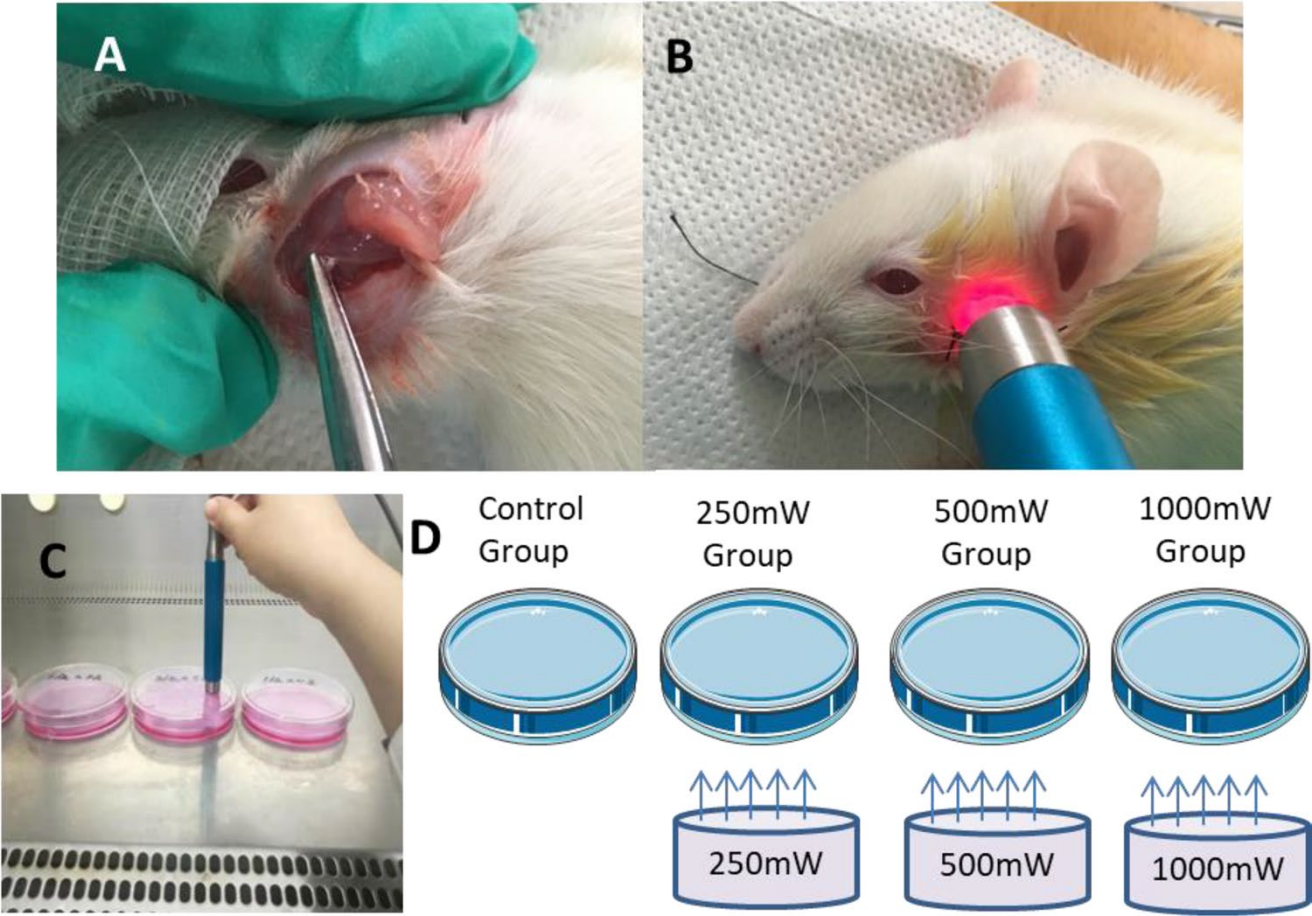
Photobiomodulation on facial nerve regeneration. **A**, crush injury was performed on the facial nerve of rats; **B**, photobiomodulation was treated at the facial nerve area after crush injury; **C** photobiomodulation was treated on SCs; **D**, diode laser irradiation of SCs with different parameters (control group; output power 250mW; output power 500 mW; output power 1000 mW irradiation for 30 s/8 h for 12 days)

All rats were equally and randomly divided into the following five groups: sham group, with natural facial nerves; control group with crushed nerve injury untreated with irradiation; 250mW group treated with an output power of 250mW, power density of 0.89 W/cm^2^, and energy density of 26.5 J/cm^2^; 500 mW group treated with an output power of 500 mW, power density of 1.77 W/cm^2^, and energy density of 53 J/cm^2^; and 1000mW Group treated with an output power of 1000 mW, power density of 3.54 W/cm^2^, and energy density of 106 J/cm^2^ (Fig. 1A, B).

### Evaluation of vibrissa movement

Vibrissa movement was graded based on five points to assess the whisking behavior of the animals: 0, no whisker movement; 1, slight whisker movement; 2, slow movement; 3, rapid movement, but different from that of contralateral normal side; and 4, symmetric movement as compared to the normal side. Evaluation of the vibrissa movement was performed at weeks 4, 8, and 12. The assay was set in a blinded fashion in order to avoid subjective bias (Table 1) (Chao et al. 2016).

**Table 1:** Evaluation of vibrissa movement

Different scores	Vibrissa movement evaluation
0	No whisker movement
1	Slight whisker movement
2	Slow movement
3	Rapid movement, but distinguishable from that of the contralateral normal side
4	Symmetric movement as compared to the normal side

### Culture of SCs and parameter of photobiomodulation

SCs (RSC96 cell lines) were obtained from Shanghai cell bank, Chinese Academy of Sciences. The cells were cultured in DMEM-F12 containing 10% fetal bovine serum (at 37 °C in a humidified incubator with 10% CO_2_) followed by incubation for 24 h to allow cell attachment prior to photobiomodulation. Photobiomodulation with continuous pulse produced by a diode laser was then applied to rats. A total of four separated groups, including control group—with no laser irradiation; 250mW group—output power of 250mW, power density of 0.89 W/cm^2^, energy density of 26.5 J/cm^2^, and irradiation for 30 s/8 h for 12 days; 500 mW group—output power of 500 mW, power density of 1.77 W/cm^2^, energy density of 53 J/cm^2^, and irradiation for 30 s/8 h for 12 days; and 1000 mW group—output power of 1000 mW, power density of 3.54 W/cm^2^, energy density of 106 J/cm^2^, and irradiation for 30 s/8 h for 12 days, were set up. The large-light-spot handle of the diode laser was perpendicularly placed to each Petri dish, and the light spot on the Petri dish followed an S-shaped track to ensure irradiation of each area (Fig. 1C, D). The medium was then supplemented with 100 μm H_2_O_2_ for another 2 h. To further evaluate the effect of PI3K/Akt activation on oxidative injury, SCs were pretreated with PI3K inhibitor LY294002 (20 μm) for 2 h before undergoing photobiomodulation (Dong et al. 2019; Sang et al. 2018).

### Proliferation, migration, and morphology of SCs

The proliferation of SCs was evaluated by seeding the cells at a density of 8.0 × 10^4^ cells/well into 6-well plates, which were then cultured by DMEM supplemented with 1% penicillin/streptomycin and 10% FBS at 37 °C in a humidified atmosphere containing 5% CO_2_. The morphology of SCs after photobiomodulation was depicted via immunofluorescence staining technology. Briefly, the samples were fixed with 4% paraformaldehyde for 24 h at 4 °C. The mouse anti-S100 (1:500, ABCAM, USA) and anti-neurofilament 200 (1:80; Sigma, USA) antibodies were applied as primary antibodies overnight at 4 °C. Secondary antibodies, including goat anti-rabbit IgG (FITC)/goat anti-mouse IgG (TRITC) (Dako Japan) and DAPI (1:200, Life Technologies), were applied for 1 h at room temperature. The images were acquired by using a laser confocal microscope (FV10i-oil, push around, Tokyo, Japan).

For real-time live-cell analysis of SCs, the cells were seeded into 96-well culture plates at a density of 3 × 10^3^ cells/ml. Cell viability was measured using an IncuCyte S3 (Essen BioScience MA, USA) at 6 h, 12 h, 18 h, 24 h, 30 h, 36 h, 42 h, 48 h, 54 h, and 60 h time points during the assay. By counting the total number of cells in each subregion and dividing it by the area of the subregion, an estimate of the local cell density in that subregion was obtained.

### Apoptosis analysis by flow cytometry

SCs after photobiomodulation in the logarithmic phase were inoculated in the culture medium at a density of 2 × 10^4^ cells/well and cultured at 37 °C for 3 days. After that, 0.25% trypsin was digested, centrifuged (1000 r/min, 5 min), and maintained in supernatant liquor. The supernatant was then discarded, and the precipitated SCs were gently mixed with 70% pre-cooled ethanol. After fixing the cells at 4 °C overnight, the SCs were re-suspended after centrifugation (1000 r/min, 5 min). Next, 25 ml propidium iodide (PI) and 20 ml RNase A were added to 1 ml of dyeing buffer solution and then were blended. Each cell sample was mixed with 0.5 ml PI staining solution. The suspended cells were homogenized, incubated at 37 °C away from light for 30 min, and observed via flow cytometry. Red fluorescence was detected at an excitation wavelength of 488 nm.

### Histomorphometric evaluation

At the end of the 12-week follow-up period, 6 rats per group were anesthetized. The facial nerve was exposed, and the facial nerve of the crush injury segment was obtained. The samples were immediately immersed in a fixation solution containing 2.5% glutaraldehyde in phosphate-buffered saline (PBS, pH 7.4) at 4 °C for 24 h. The distal portion (5 mm distal to the injury) was used for immunofluorescence and histopathology evaluation. The 20–30 μm thick cross sections were cut by a freezing microtome. The mouse anti-S100 (1:500, ABCAM, USA) and anti-neurofilament 200 (1:80; Sigma, USA) primary antibodies were applied and incubated overnight at 4 °C. The goat anti-rabbit IgG (FITC) and anti-mouse IgG (TRITC) secondary antibodies (Dako Japan) were applied for 1 h at room temperature. The images were then acquired under a confocal laser microscope (FV10i-oil, push around, Tokyo, Japan), and were digitally recorded and processed by Image-Pro Plus.

The mean fiber density was calculated by dividing the total number of myelinated nerve fibers within the sampling field by its area (N/mm^2^). To assess myelin thickness, ultrathin sections were obtained by the same ultra-microtome (Leica, Ultracut) and double-stained with uranyl acetate and lead citrate. The sections were analyzed with a transmission electron microscope (TEM, JSM 1200 IIEX, JEOL, Tokyo, Japan). After imaging of myelin, OPTIMAS Ver. 6.5 (Media Cybernetics, Bethesda, MD) was used to measure myelin thickness.

### Retrograde nerve labeling

Retrograde labeling and counting of back-labeled sensory neurons were performed as previously described (Li et al. 2012). The retrograde labeling of the facial nerve sample with 1,1’-dioctadecyl-3,3′3’-tetramethylindocarbocyanine perchlorate (Dil, C59H97ClN2O4, Molecular Probes; Eugene, OR, USA) was used for measuring nerve regeneration after photobiomodulation. Five days after Dil injection in the facial nerve, rats were rapidly anesthetized using an intraperitoneal injection of chloropent (3 ml/kg), and transcardially perfused using a 0.9% saline solution with 1% heparin (JW Pharmaceutical, Seoul, Korea) followed by 4% paraformaldehyde (Merck, Darmstadt, Germany) in 0.1 M sodium phosphate buffer (pH7.2). Immediately after fixation, the facial nuclei were extracted and post-fixed with the same fixative at 4 °C for 24 h. To prevent the occurrence of freezing artifacts during the preparation of frozen tissue sections, the tissues were fixed in 30% sucrose (Sigma, St, Louis, MO, USA) at 4 °C for 24 h. The tissue sections were observed using a confocal laser-scanning microscope (CLSM, LSM700, Carl Zeiss, Oberkochen, Germany). The number of Dil back-labeled neurons in each facial nuclei was counted, and the means were then compared among the groups. The labeled neurons at each facial nuclei section were randomly selected, and the soma size of the neuron was measured and averaged with computer software (OPTIMAS Ver. 6.5) according to the technique reported by Li et al.

### Western blot analysis

Facial nerve protein was extracted, and western blot analysis and quantification were performed as previously described (Sang et al. 2018). All proteins were extracted via Trizol (peqGold TriFast, Peqlab), precipitated from the organic phase using ethanol, and pelleted by centrifugation (12,000* g*, 10 min, and 4 °C). The protein pellets were washed three times with a combination of 5% 0.3 mol/l guanidine hydrochloride (Sigma-Aldrich) and 95% ethanol and once with 100% ethanol (Merck) and followed by centrifugation (7500* g*, 5 min, 4 °C). The supernatants were discarded and the dry protein pellets were solubilized in analytical grade water with 1% sodium dodecyl sulfate (SDS, Sigma-Aldrich) in it. The protein was equally separated (up to 3 μg/lane) on a gel with 12% SDS–polyacrylamide and blotted onto a nitrocellulose membrane. The membranes were blocked with 5% skimmed milk in Tris-buffered saline for 120 min, incubated with primary antibodies, and diluted in 5% bovine serum albumin (Sigma-Aldrich) in TBS-T in a roll mixer at 4 °C for 12 h. The antibodies included NGF (1:1000), BDNF (1:1000), p-Akt (1:1000), Akt (1:1000), Nrf2 (1:1000), Bcl-2 (1:1000), Bax (1:1000), and GAPDH (1:10,000). The membrane was washed twice with TBS-T, incubated in 1 × New Blot IR Stripping Buffer (LI-COR Biosciences) on a shaker at room temperature for 5 min, and washed thrice in PBS, and the membranes were then reproved with total antibodies. The ratio of analyzed protein to housekeeping gene α-tubulin was analyzed with densitometry by Image Studio Version 5.0.21 (LICOR Biosciences).

### Quantification of NGF, p75NTR, and trkA mRNA expression by real-time PCR

Twelve weeks after surgery, 3 animals per group were anesthetized, and the facial nucleus (FNs) of the left side was obtained. Total RNA was extracted with Trizol reagent (Invitrogen, Carlsbad, CA, USA), and reverse transcribed into cDNA by first-strand synthesis kit (Invitrogen). The amount of cDNA was counted by real-time PCR. The following primers were used to replicate specific cDNA segments: NGF (GenBank Reference Sequence no: XM_227523.3), p75NTR (GenBank Reference Sequence no. X05137.1), trkA (GenBank Reference Sequence no. M85214.1), and glyceraldehyde-3-phosphate dehydrogenase (GAPDH) (GenBank Reference Sequence no: NM_017008.3) (Table 2). GAPDH was used as an internal control for normalization. The relative mRNA levels were derived by the cycle threshold (△Ct) method as previously described (Applied Biosystems Manual, Foster City, CA). The PCR reactions were assessed at least twice (Li et al. 2012).

**Table 2:** Primer sequences

Gene	Sequence(5’-3’)	mRNA position (start)	mRNA position (end)	Product size (bp)
NGF	Forward: AAG ACC ACA GCC ACG GAC AT Reverse: CGC CTT GAC AAA GGT GTG AG	722	916	195
p75^NTR^	Forward: TGG CGG ATA TGG TGA CCA CT Reverse: GCA GCT GTT CCA CCT CTT GA	770	921	152
TrkA	Forward: AGC CGT GGA ACA GCA TCA CT Reverse: CGC ATG GTC TCA TTG GTC AG	943	1095	153
GAPDH	Forward: GCA TCC TGC ACC AAC T Reverse: GCA GTG ATG GCA TGG ACT GT	802	902	101

### Statistical analysis

All measurements and histological observations were carried out in a blinded fashion. The measurement data were expressed as means ± standard deviation. A one-way analysis of variance (ANOVA) was used for comparing multiple groups, followed by Tukey’s test. Fisher’s exact test was used for comparing mortality among groups. A P-value of ≤ 0.05 was considered statistically significant. All data were examined using SPSS 21.0 statistical software (SPSS Inc., Chicago, IL, USA).

## Results

### Photobiomodulation improves facial nerve regeneration

**Fig. 2:**
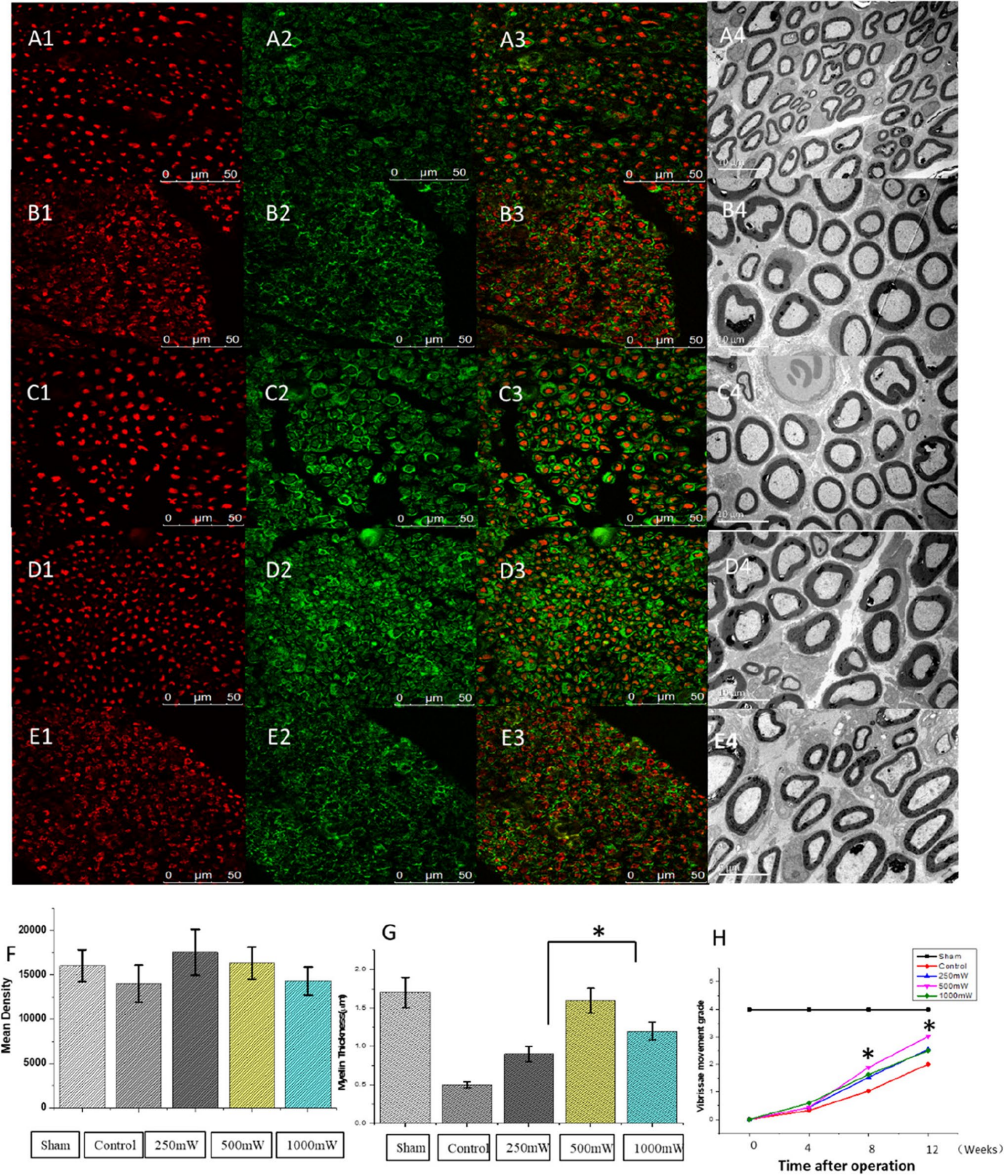
Photobiomodulation improves facial nerve regeneration. NF-200 (red) and S-100 (green) in cross sections of facial nerves at week 12 after the operation. Sham group (A1–A3), control group (B1–B3), 250mW group (C1–C3), 500mW group (D1–D3), and 1000mW group (E1–E3). Scale bar: 50 μm. Electron microscopic analysis in cross sections of facial nerves at week 12 after operation in groups: A4, sham group; B4, control group; C4, 250mW group; D4, 500mW group; and E4,1000mW. The presence of more regenerated myelin nerve fibers in 250mW and 500mW groups. F, quantitative analysis of the mean density of fluorescence intensity. (*p < 0.05) G, quantitative analysis of myelin sheath thickness. Scale bar: 10 μm. H, vibrissa movement grade. The vibrissa movement was assessed at weeks 4, 8, and 12 after treatment with photobiomodulation. The vibrissae movement in the 500mW group was faster than in the control groups at weeks 8 and 12 (p < 0.05)

Double immunohistochemistry with anti-NF200 and anti-S100b of injured facial nerves was performed 12 weeks after surgery. Interestingly, S100 positive (green) myelin sheaths surrounded NF200 positive (red) axons were observed in all cross sections. The myelin sheaths were thicker in the 250mW and 500mW groups than those in the control group. In addition, stronger axonal regeneration and larger myelin sheath coverage were found in 250mW and 500mW groups than in the crush group (Fig. 2A1–E3). The density of immunopositive areas of both NF200 and S100 in the 250mW and 500mW groups was greatly increased in the crush group and was similar to the normal nerve tissues (Fig. 2F) (normal group: 16,001 ± 1809mm^2^; crush group: 14,000 ± 2100 mm^2^; 250mW group: 17,530 ± 2600 mm^2^; 500mW group: 16,331 ± 1800 mm^2^; and 1000mW group: 14,300 ± 1600 mm^2^).

TEM was used to evaluate the degree of denervation, revealing a deficit in the myelinated fiber population in the crush group compared to other laser groups (Fig. 2A4–E4). Increased myelinated fibers were observed in both 250mW and 500mW groups when compared to the crush group (Fig. 2G). Regenerated myelinated fibers in the 500mW group showed well-regulation in micro-fascicles, which was similar to that of normal nerve fibers and somewhat greater than those in the crush group. It is widely recognized that axon counting and myelin thickness are crucial for assessing axonal myelination. The myelin thickness was significantly (P < 0.05) higher in both 250mW and 500mW groups compared to the laser group.

On day 1 of operation, all rats were deprived of their ability to whisk, and later the function of vibrissae movement was gradually recovered. At week 4 after laser therapy, the degree of vibrissa movement was a little higher in the 1000mW group than in other groups, but the difference was insignificant. At weeks 8 and 12 after surgery, the average vibrissae movement grade of rats in all laser groups was higher compared to the control group. In particular, the grade of the 500mW group was significantly higher compared to the control group at weeks 8 and 12 (Fig. 2H).

### Nerve regeneration in the facial nucleus

**Fig. 3:**
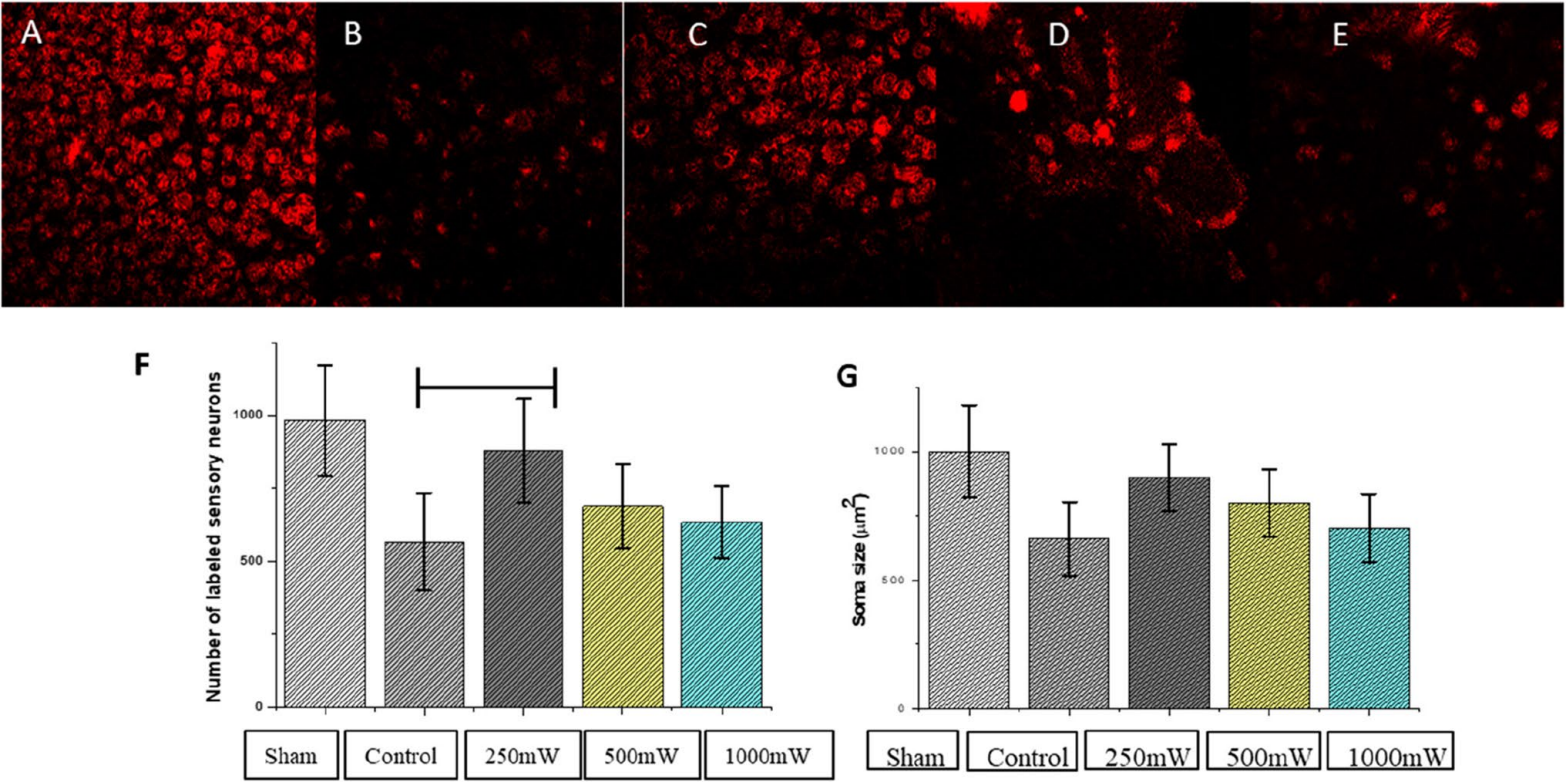
Retrograde nerve labeling in facial nucleus. Dil staining was performed to investigate labeled neurons in the facial nucleus (sham group (**A**), control group (**B**), 250mW group (**C**), 500mW group (**D**), and 1000mW group (**E**)) in retrograde. The 250mW group showed improved recovery, and the number of retrograde-labeled neurons (**F**) and soma size of the facial nucleus (**G**) were larger compared with the crush injury groups) (p < 0.05)

After photobiomodulation treatment on rats with crushed injury facial nerve, Dil staining was performed to investigate the retrogradely labeled neurons in the facial nucleus (Fig. 3A–E). The mean number of labeled neurons in the control group and 250mW group was significantly greater than that in the crushed injury group (Fig. 3F). The size of soma in the labeled neuron remained the largest in the 250mW group when compared to other groups (Fig. 3G) without statistical significance.

### Photobiomodulation increases functional protein secretion and mRNA levels

**Fig. 4:**
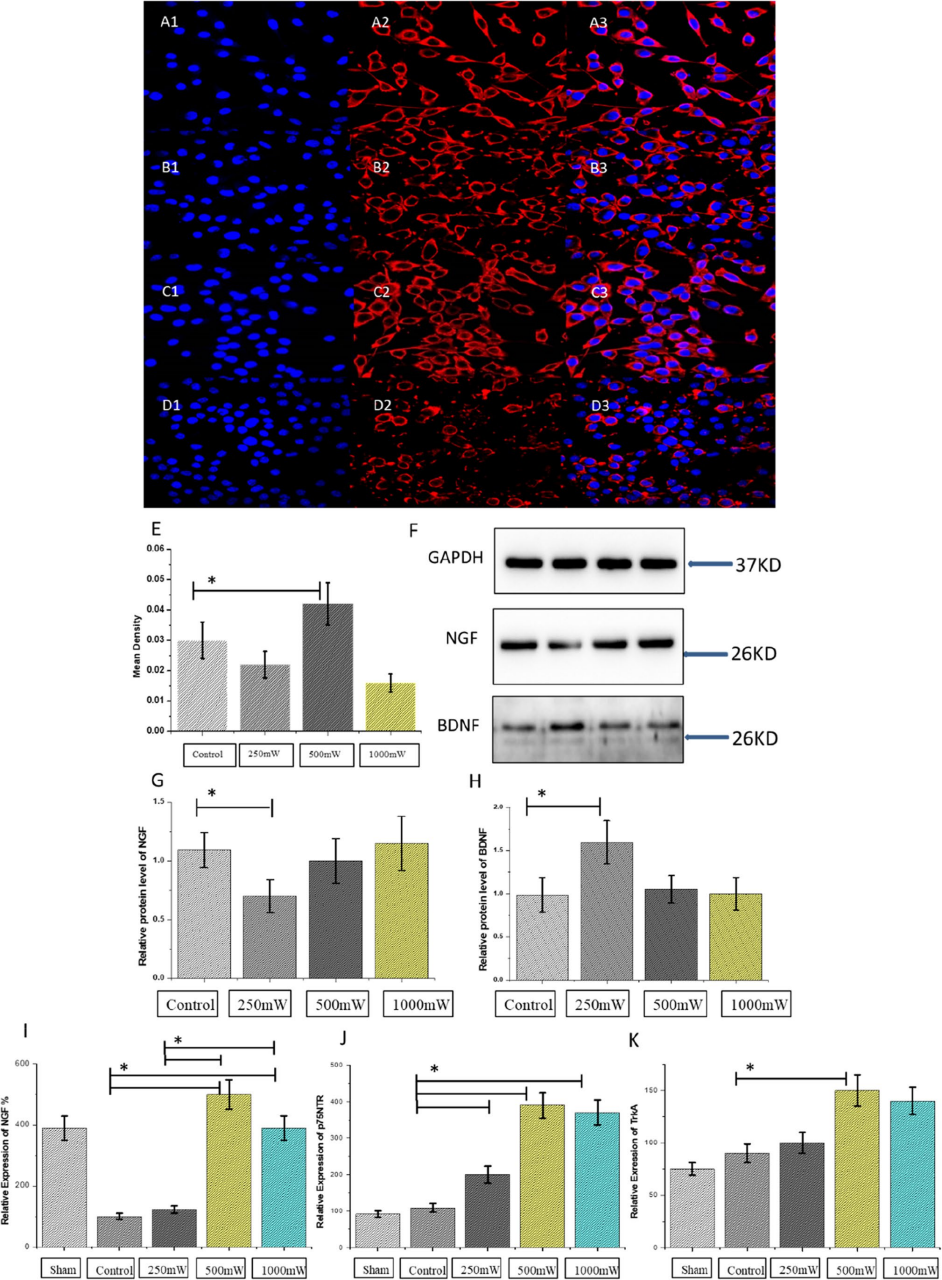
Immunofluorescence images of SCs treated with photobiomodulation for 12 days. The S100 marker of SCs was stained in red, and the nuclei were stained by DAPI in blue (A1–D3). Scale bar = 100 μm. E, quantitative analysis of the mean density of S100 fluorescence intensity (control&500mW, *p < 0.05). F, western blotting of protein levels of NGF and BDNF in SCs irradiated by diode level laser (control&500mW, *p < 0.05). G–H, quantification analysis of NGF and BDNF expression. I–K, quantitative real-time polymerase chain reaction (relative expression, %) of NGF, p75NTR, and TrkA mRNA expression levels in the facial nerve at week 12 after operation (**P < 0.01; *P < 0.05)

The morphological observation under an inverted optical microscope showed that SCs after photobiomodulation in 250mW and 500mW had healthy growth and adhered to the wall, resembling the spindle-shaped willow leaves, which was similar to that in the control group. Compared with the control group (without laser irradiation), SCs in the 500mW group demonstrated the highest cell density under a microscope (Fig. 4A1–D3). The mean fluorescence intensity of S100 in the 500mW group was increased and was the highest when compared to all other groups, and significantly higher than the control group (Fig. 4E).

Western blot analysis performed with photodensitometry is presented in Fig. 4F. Western blot analysis of SCs protein lysate demonstrated the highest level of nerve growth factor (NGF) in 1000mW group without any significant difference. Furthermore, the highest level of bone-derived neurotrophic factor (BDNF) in the 250mW group was significantly different compared to the control group (Fig. 4F) (*P < 0.05).

Quantitative RT-PCR of NGF, p75NTR, and trkA expression is shown in Fig. 4I–K, and the results revealed that the levels of all three markers were significantly highest in the 500mW group (**P < 0.01). The mRNA expression levels of TrkA and p75NTR in the 1000mW group were also significantly higher compared to the control group (*P < 0.05).

### The SCs viability by IncuCyte

**Fig. 5:**
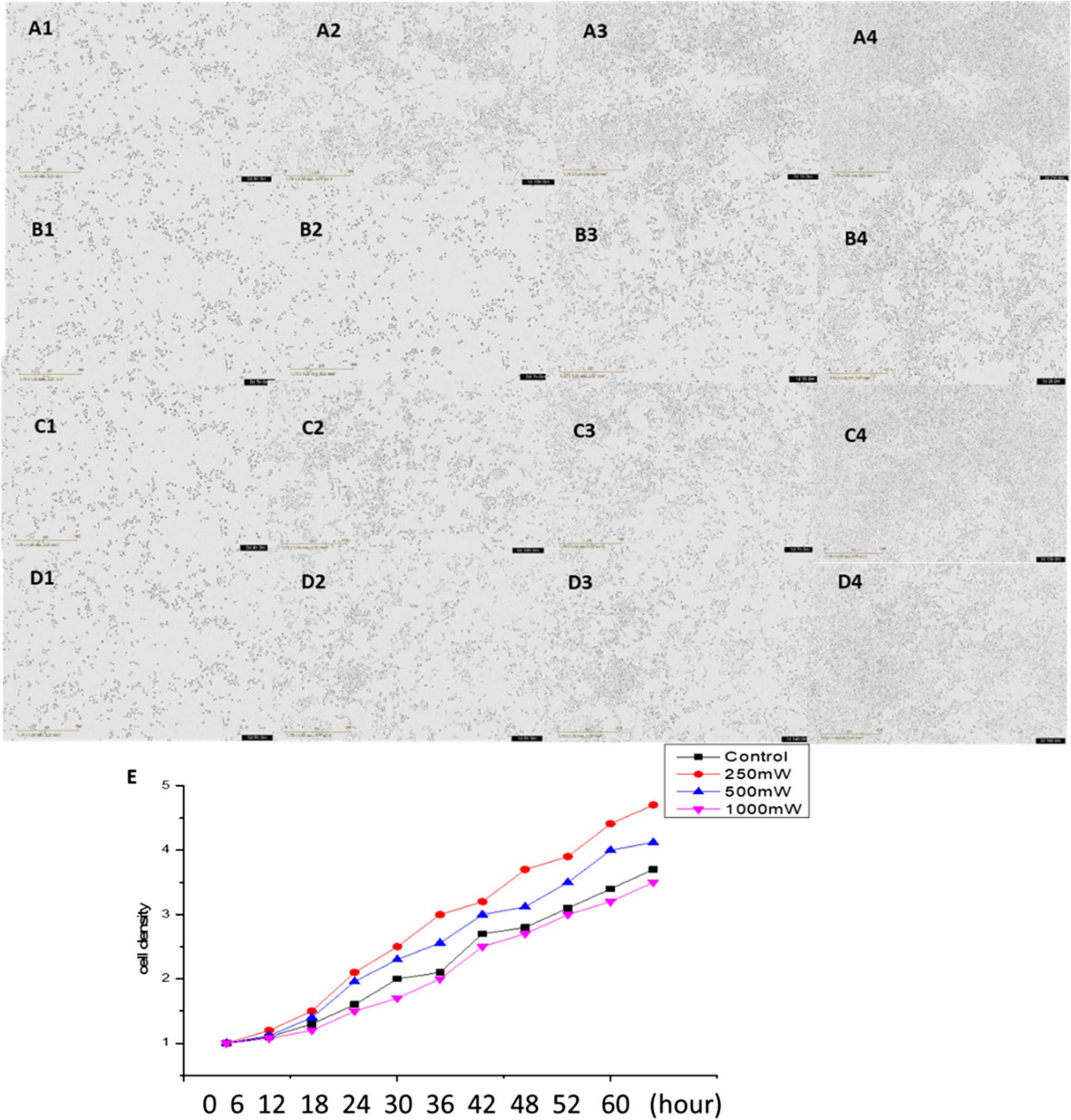
SCs proliferation by IncuCyte. The real-time live SCs evolution of an IncuCyte assay. Images taken after (a) 0, (b) 8, (c) 16, (d) 24, and (e) 46 h after the scratch was performed (A, control; B**, **250mW; C, 500mW; D, 1000mW). The scale bar corresponds to 300 μm. F, comparison of the average experimental cell density C(t) (crosses) and the logistic growth curve using our estimates of K and λ (solid)

The real-time live-cell viability was analyzed using the Incucyte. To check the effects of photobiomodulation on the cell viability in SCs. SCs were seeded in a 96-well plate. Data were analyzed in three subregions within each IncuCyte S3 image at 6 h, 12 h, 18 h, 24 h, 30 h, 36 h, 42 h, 48 h, 54 h, and 60 h time points during the assay (Fig. 5A1–D4). Figure 5 shows the time evolution of the average cell density, calculated by averaging the six estimates of cell density from each subregion at each time point. The viability of all the SCs in each group gradually increased with the incubation time. The cell viability in the 250mW group was higher than in other groups.

### Photobiomodulation inhibits apoptosis in vitro

**Fig. 6:**
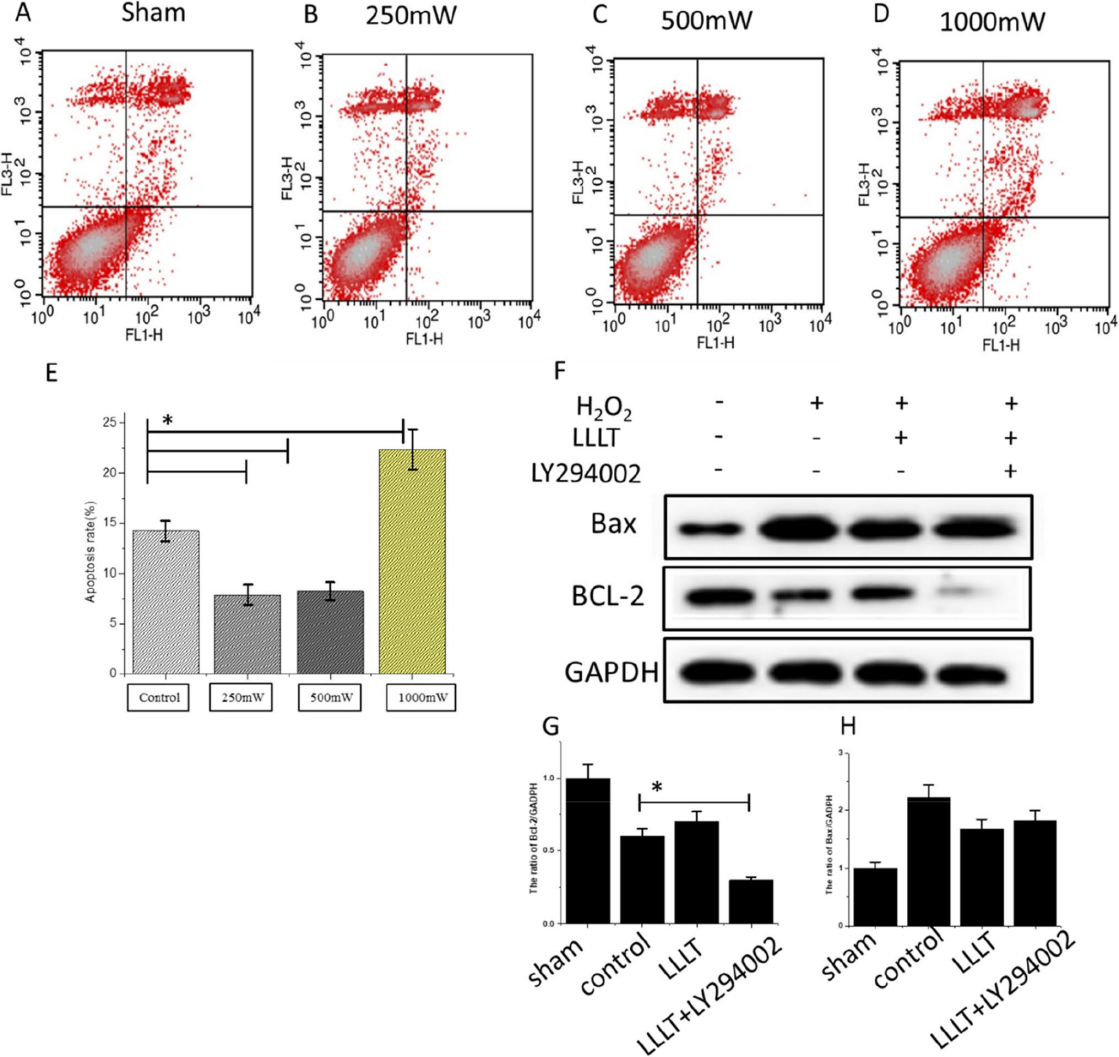
Photobiomodulation inhibits SCs apoptosis in vitro. **A–E**, apoptosis of SCs treated with photobiomodulation was analyzed by Annexin V and PI staining. The apoptotic rate in 250mW and 500mW groups was significantly decreased, while in 1000 mW groups it was significantly increased after treatment with photobiomodulation. Data were presented as means ± SD values. *P < 0.05 **F**, the protein levels of Bax and Bcl-2 were detected by western blotting; **G–H**, quantification analysis of Bax and Bcl-2 expression. *P < 0.05

As shown in Fig. 6, apoptosis of SCs treated with photobiomodulation was analyzed by annexin V and PI staining. The results of flow cytometry analysis showed that the apoptotic rate was significantly decreased in the 250mW group (7.91%) and in the 500mW group (8.27%) compared to the control group (14.24%). However, the apoptotic rate of 1000mW group was significantly higher than that of the control group (Fig. 6A–E) (p > 0.05). This suggested that photobiomodulation can inhibit apoptosis of SCs at the parameter of 250mW and 500mW.

Western blotting was used for further detection of apoptosis of SCs caused by H_2_O_2_. The results showed down-regulation of Bax and up-regulation of Bal-2 levels in photobiomodulation-treated SCs when compared with H_2_O_2_-treated SCs. The results suggested that photobiomodulation has anti-oxidative effects in reducing apoptosis (Fig. 6F–H). Treatment with PI3K inhibitor LY294002 alleviated the anti-apoptotic effects of photobiomodulation on H_2_O_2_-treated SCs.

### Photobiomodulation alleviates oxidative injury and improves axon regeneration via PI3k/Akt signaling in vitro and in vivo

**Fig. 7:**
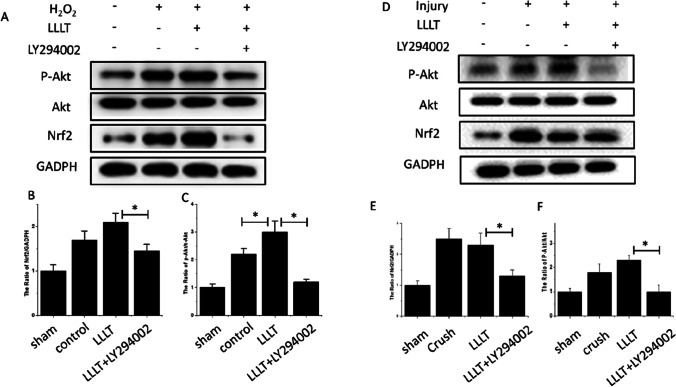
Photobiomodulation reduces SC apoptosis and improves facial nerve regeneration through PI3K/AKT signaling pathway. **A**–**C**, the protein expression of p-Akt, Akt, and Nrf2 in SCs were detected by western blotting. **D–F**, the protein expressions of p-Akt, Akt, and Nrf2 in the facial nerve were detected by western blotting. In vivo and In vitro western blotting results show photobiomodulation improves the facial nerve regeneration through the PI3K/AKT signaling pathway. *P < 0.05

To investigate the molecular mechanism by which photobiomodulation protects SCs against H_2_O_2_-induced apoptosis in vitro, the Nrf2, p-Akt, and Akt protein levels were examined by western blotting analysis. The levels of Akt phosphorylation were slightly higher than in the H_2_O_2_ group, and even higher in photobiomodulation-treated group, which was in accordance with the p-Akt/Akt ratio. The up-regulation was reversed by treatment with LY294002. The Nrf2 protein levels showed a similar trend. These results indicated that the activation of PI3K/Akt signaling contributed to photobiomodulation-treated facial nerve regeneration (Fig. 7A–F).

## Discussion

Many studies have proved that diode laser biological stimulation can promote axonal regeneration and functional recovery (Yazdani et al. 2012; Kouhkheil et al. 2018; Takhtfooladi et al. 2015), which was furthermore confirmed by our study. Although the mechanisms of promotion of nerve regeneration by diode laser biological stimulation still remain unclear, it is believed that the promotion of migration, proliferation, and development of SCs and increased secretion of SCs in a large number of NGF factors that promote axonal extension have a relevant role. Laser treatment can cause the formation of microtubule and microfilament protein in axons chemotactically (Yazdani et al. 2012; Van Breugel and Bar 2013) and promote vascularization of peripheral nerve regeneration (Masoumipoor et al. 2014).

Recent studies have shown that SCs, the principal glial cells of the PNS, secrete neurotrophic factors that promote the regeneration of the peripheral nerve. Photobiomodulation stimulates the proliferation of SCs in vitro. Many studies have reported that irradiation ranging from 632 to 901 nm enhances nerve cell differentiation, proliferation, axonal growth and myelination, and improves morphological recovery in experimental sciatic nervous lesions in a dose-dependent manner (Pouriran et al. 2016; Sefati et al. 2018; Dias et al. 2019). In our study, the apoptotic rate of SCs in 250mW and 500mW groups was lower than that in the control group, but the rate of apoptosis in the 1000mW group was higher than that of the control group, thus suggesting that photobiomodulation with the parameters of 250mW and 500 W could restrain apoptosis of SCs. In real-time live-cell analysis, the proliferation rate of SCs in 250mW and 500mW groups was higher than that of the control group and 1000mW group, indicating that photobiomodulation in 250mW and 500mW group improved the proliferation of SCs. Immunocytochemistry with anti-S100β and DAPI that served as markers of SCs showed a marked enhancement in purity after the cells were sorted, almost reaching 99% purity. In addition, western blots also showed a steady increase of NGF in the 500mW group, while a much higher level of BDNF was observed in the 250mW group.

While the most effective laser application with regard to wavelength, energy density, continuous or pulsed mode, treatment duration time, and the wave is still controversial, the use of laser treatment definitely facilitates nerve regeneration (Kouhkheil et al. 2018; Pol et al. 2016). Our study assessed morphological changes using TEM, showing thicker myelin sheaths with denser alignments in 250mW and 500mW groups. Many studies have assessed functional recovery, histological and micro-morphological changes, and electrophysiological improvement after the introduction of photobiomodulation, which proved to have beneficial effects on the regeneration of rats’ sciatic nerve injury (Kouhkheil et al. 2018; Pol et al. 2016; Mashhoudi Barez et al. 2017). Wang et al. (2014) have reported that an 808-nm laser treatment with energy density (3 J/cm^2^ and 8 J/cm^2^) is capable of enhancing sciatic nerve regeneration after a crush injury. Buchaim et al. (2015) have reported that laser treatment (wavelength of 830 nm, 30 mW optical power output of potency, and energy density of 6 J/cm^2^) has satisfactory results on facial nerve regeneration. The irradiation parameters they described were different from ours, where we showed that photobiomodulation of 980 nm performed on day 1 after surgery and during the postoperative period (three times weekly for a total of 5 weeks) at output power (250mW, 500mW, and 1000mW) accelerated functional recovery and enhanced facial nerve regeneration in crush injury.

Several lines of evidence have implicated the involvement of PI3K/Akt signaling in modifying the survival, growth, splitting, proliferation, and apoptosis of SCs (Huang et al. 2017; Sang et al. 2018). Additionally, PI3K/Akt signaling pathway has a critical survival factor role in repairing and regeneration processes after nerve injury (Sang et al. 2018). Activation of PI3K/Akt signaling blocks the mitochondrial apoptotic pathway, while inhibition of PI3K/Akt signaling activates the apoptotic pathway (Huang et al. 2017; Mashhoudi Barez et al. 2017). In this study, the photobiomodulation in 250mW improved phosphorylation of Akt through PI3K activation and prevented apoptosis of SCs induced by inhibiting Akt phosphorylation. Following Akt phosphorylation, the activation of the PI3K/Akt signaling pathway was mediated by Bcl-2 phosphorylation, and subsequent inhibition of Bcl-2 and BAD phosphorylation (Andraus et al. 2017; Chang et al. 2017). Phosphorylated BAD then binds to 14–3-3 protein, releasing Bcl-xL into the cytoplasm (Dong et al. 2019; Andraus et al. 2017; Chang et al. 2017). The release of Bcl-xL, in turn, unblocks the translocation of Bax protein into the mitochondria, resulting in disruption of mitochondrial membrane potential (MMP).

Nrf2 is a vital regulator of oxidative damage, neural protective functioning, and inflammation modulation that are involved in the traumatic brain injury models (Dong et al. 2019; Wu et al. 2016). In this study, the protein levels of Nrf2 were slightly higher in the H_2_O_2_ group, and were further higher in photobiomodulation-treated group, while treatment with LY294002 reversed these effects. Oxidative stress activity decreased anti-apoptotic protein Bcl-2 and increased the expression of pro-apoptotic protein Bax, severely influencing cell survival and inducing cell apoptosis (Huang et al. 2017; Dong et al. 2019; Sang et al. 2018). In our study, the protein levels of Bcl-2 were up-regulated, but the protein levels of Bax were down-regulated after oxidative damage treated with photobiomodulation, while this effect was partially reversed by a combination of photobiomodulation and LY294002. This result indicated that oxidative apoptosis after peripheral nerve injury was probably regulated by PI3K/Akt signaling. In our study, the expression levels of phosphorylated p-Akt/Akt and Nrf2 and the dimerization of Bax/Bcl-2 level were assessed in regular photobiomodulation used to treat crushed facial nerve. In addition, the effect of photobiomodulation in 250mW combined with PI3K/Akt pathway inhibitor (LY294002) was examined in SCs in vitro, which indicated that apoptosis of SCs occurs through down-regulation of PI3K/Akt signaling in vitro.

To test whether photobiomodulation treatment inhibits crushed-induced oxidative stress in the facial nerve via PI3K/Akt, the expression of oxidative stress–related proteins Nrf2, P-Akt, and Akt was detected by western blotting. The level of Nrf2 was higher in the injury group and lower in the injury-added-with-LY29400 group. The ratio of P-Akt/Akt was higher in the injury group and lower in the injury-added-with-LY29400 group. The levels of Akt phosphorylation were slightly increased after PNI, and these levels were even higher after PNI rats received photobiomodulation treatment, which was also confirmed by the p -Akt/Akt ratio. These results suggest that photobiomodulation in 250mW induces mitochondrial-dependent apoptosis by down-regulation of PI3K/Akt signaling in the rat facial nerve in vivo. Furthermore, PI3K-Akt activation mainly contributed to neuroprotection through post-conditioning treatment (Dong et al. 2019; Sang et al. 2018).

This paper has some limitations. In this study, crush injury was used as an in vivo injury model according to the previous literature. Other studies used the feedback-controlled electronic squeezer method, which is considered to be more objective in recording the degree of damage. Furthermore, hydrogen peroxide-damaged SCs were used as in vitro injury models according to the previous literature. During the process of peripheral nerve regeneration and repair, the early stage (4-week time point) data remained unstable, while the overall trend of 12 treatment at week 8 showed the best treatment effect in the 250mW group, which was similar to the previous study (Lv et al. 2017). We only checked the energy density of 16.71 J/cm^2^ on a facial nerve in crush injury. To investigate the molecular mechanism, we only focused on the effective results of the pathway of PI3K/AKT in the facial crush nerve and did not address the negative effects on the normal, natural nerve. We used the photobiomodulation on the crushed facial nerve from the day after surgery till the postoperative period 3 times per week for 5 consecutive weeks; however, the observation and evaluation time lasted 12 weeks.

## Conclusion

Based on the results of the present study, the application of a laser source at a 980-nm wavelength and at doses of 250mW and 500mW to an injured rat facial nerve immediately after crush injury had a beneficial effect on facial nerve regeneration, including better functional recovery and morphological changes. In addition, the neuroprotective function of photobiomodulation was associated with excessive suppression of oxidative stress–induced SCs apoptosis via activation of the PI3K/Akt signaling pathway. These results indicated that photobiomodulation might be regarded as a potential therapeutic agent for peripheral nerve reconstruction after injury.
